# Supplementary material for: A comparison of ImageJ and machine learning based image analysis methods to measure cassava bacterial blight disease severity
Source: Plant Methods. 2022 Jun 21;18:86. doi: 10.1186/s13007-022-00906-x (PMC9210806; doi:10.1186/s13007-022-00906-x)
Supplement: Supplementary file 1 — Additional file 1: Movie S1. Movie example of ImageJ based analysis method. [file 13007_2022_906_MOESM1_ESM.docx]

**Additional File 1: Movie example of ImageJ based analysis method** Available for download on figshare or online at <https://youtu.be/EtEzRls4Jh4>
