# Supplementary material for: A comparison of ImageJ and machine learning based image analysis methods to measure cassava bacterial blight disease severity
Source: Plant Methods. 2022 Jun 21;18:86. doi: 10.1186/s13007-022-00906-x (PMC9210806; doi:10.1186/s13007-022-00906-x)
Supplement: Supplementary file 2 — Additional file 2: Table S1. Machine learning tool commands. A table of the command syntax, function, and description of inputs and outputs for each command. [file 13007_2022_906_MOESM2_ESM.pdf]

Tool Commands:	Function:	Inputs Needed:	Outputs:
ML_CREATE	Create .YAML classifier file based on of Naïve Bayes or Support Vector Machine learning algorithms	Representative RGB (input) and mask (labeled) images	.YAML file
ML_PRED	Create prediction image of what pixels will be classified as object of interest based on classifier file	Classifier file, input image, and type of machine learning method used to create classifier file	Prediction .PNG file
ML_PROC	Process all images using appropriate classifier type	Classifier file, input image, and type of machine learning method used to create classifier file, size of ROI selection box	Gray corrected, color-map and prediction .PNG file for each image and .txt files with shape and color data for each object
ML_STAT	Create summary statistics of classifier file accuracy rate	Classifier file, input image, and type of machine learning method used to create classifier file	Summary true positive, false positive, false negative, true negative, precision, recall, accuracy, true positive rate, false positive rate, true negative rate, and false discovery rate statistics for the classifier file
