# Supplementary material for: A comparison of ImageJ and machine learning based image analysis methods to measure cassava bacterial blight disease severity
Source: Plant Methods. 2022 Jun 21;18:86. doi: 10.1186/s13007-022-00906-x (PMC9210806; doi:10.1186/s13007-022-00906-x)
Supplement: Supplementary file 3 — Additional file 3: Movie S2. Movie example of machine learning based analysis method. [file 13007_2022_906_MOESM3_ESM.docx]

**Additional File 3: Movie example of machine learning based analysis method** Available for download on figshare or online at <https://youtu.be/Dw2VebjExZw>
