# Supplementary material for: A comparison of ImageJ and machine learning based image analysis methods to measure cassava bacterial blight disease severity
Source: Plant Methods. 2022 Jun 21;18:86. doi: 10.1186/s13007-022-00906-x (PMC9210806; doi:10.1186/s13007-022-00906-x)
Supplement: Supplementary file 4 — Additional file 4: Table S2. Machine learning measurement types. A table of measurements generated from the machine learning tool and their descriptions. [file 13007_2022_906_MOESM4_ESM.pdf]

Measurement type:	Description:
Area	Area of object
Hull_area	Area of convex hull (shape of the smallest convex set)
Solidity	Ratio: area / convex hull area
Perimeter	Perimeter around object
Width	Width of object
Height	Height of object
Cmx	Center of mass x: position of center of mass
Cmy	Center of mass y: position of center of mass
Hull_verticies	Number of convex hull vertices
Ex	Ellipse center x: position of the center of the minimum bounding ellipse
Ey	Ellipse center y: position of the center of the minimum bounding ellipse
E-major	Ellipse major axis: length of the major axis of the minimum bounding ellipse
E-minor	Ellipse minor axis: length of the minor axis of the minimum bounding ellipse
E-angle	Ellipse angle: angle of rotation of the bounding ellipse major axis
Eccen	Ellipse eccentricity: eccentricity of the bounding ellipse
Circ	Circularity
round	Roundness
Ar	Aspect ratio
Fd	Box count fractal dimension
Gray scale	Lightness channel histogram from 0-255
